# A Crossover Study From a Gender Perspective: The Relationship Between Job Insecurity, Job Satisfaction, and Partners’ Family Life Satisfaction

**DOI:** 10.3389/fpsyg.2018.01481

**Published:** 2018-08-15

**Authors:** Federica Emanuel, Monica Molino, Alessandro Lo Presti, Paola Spagnoli, Chiara Ghislieri

**Affiliations:** ^1^Department of Psychology, University of Turin, Turin, Italy; ^2^Department of Psychology, University of Campania “Luigi Vanvitelli", Caserta, Italy

**Keywords:** job insecurity, job satisfaction, family life satisfaction, crossover, spillover, permanent workers

## Abstract

**Background:** In the last years, many changes have involved the labor context: new ways of working, more flexibility and uncertainty, new and more insecure job contracts. In this framework, perceived job insecurity, worker’s perception about potential involuntary job loss, has received renewed interest, also for those workers with a permanent contract in Italy. Consequences of job insecurity on work-related outcomes such as job satisfaction have been demonstrated; nevertheless, its possible effects outside the workplace seem to be underestimated so far. Moreover, literature highlighted the importance to consider gender as a possible moderator in the relationship between one partner’s stressors and the other partner’s strain.

**Aim:** According to spillover and crossover theories, this study aim was to investigate the relationship between job insecurity and family life satisfaction of both partners, through the mediation of job satisfaction. The model has been simultaneously tested in two groups, women and men, in a sample of permanent workers.

**Method:** The research involved a convenience sample of 344 employees with permanent contract (53% female) from different occupational sectors. Participants (focal persons) and their partners filled out a self-report questionnaire.

**Results:** The multi-group SEM indicated a full mediation of job satisfaction in the relationship between job insecurity on the one side, and both individual’s and his/her partner’s family life satisfaction on the other side in both groups.

**Conclusion:** These study findings highlighted how job insecurity may be indirectly and negatively related to both members’ family life satisfaction, through the mediation of job satisfaction. As regards gender, similar spillover and crossover patterns emerged, contributing to that literature that highlights a greater similarity in the models of interaction between work and family among women and men. Interventions should be aimed at reducing perception of job insecurity among workers, including those with permanent contract. Employers should improve communication and flow of information about future organizational changes. Moreover, interventions useful to monitor and reinforce employees’ job satisfaction should be planned. Finally, career practitioners may provide counseling and coaching projects aimed at strengthening employees’ employability and their ability to deal with changes.

## Introduction

Over the past years, many Western countries have passed through a period of economic slowdown and occupational uncertainty that triggered rapid changes in the working world (Direnzo and Greenhaus, 2011; Vostal, 2014). In this scenario, employment uncertainty increased, and the rise of sharing economy, the development of digital and technological tools, the shift toward new ways of working, the higher levels of flexibility supported by new job contracts suggest that job insecurity will persist in being an important characteristic in workers’ lives. In Italy in 2015, in the frame of the so called “Jobs Act" (Law n. 183/2014), the legislative decree n. 23 introduced a new type of permanent employment contract with rising protections against unfair dismissal (“Contratto a Tutele Crescenti"). This type of contract, which implies permanent employment, restricted the reintegration possibilities for workers in the event of unlawful dismissal, inducing a perception of lower job stability and security than before.

It is in this perspective that job insecurity has been receiving a significant amount of interest from researchers in recent years (Lee et al., 2018). Several reviews summarized its consequences in the work domain, including job satisfaction (Sverke et al., 2002; Cheng and Chan, 2008; Keim et al., 2014; De Witte et al., 2016). Nevertheless, possible effects of job insecurity outside the workplace seem to be underestimated (Sora and Höge, 2014) and research on the family domain is still looking for univocal and coherent evidence.

Differences between permanent and temporary workers have been investigated (e.g., Silla et al., 2005), with temporary workers showing higher levels of perceived job insecurity and related consequences. However, changes in labor market, working cultures and types of employment contract indicate that also permanent workers may be worried about their job continuity, an issue that needs to be addressed in Italy. For these reasons, supported by spillover and crossover theories, this study aims at understanding whether job insecurity spills over into family-related outcomes and crosses over from the employee to his/her partner, considering job satisfaction as a potential mediator. Specifically, the study investigated the relationship between one worker’s perceived job insecurity and family life satisfaction of both the person and his/her partner, through the mediation of job satisfaction of the person. These relationships have been explored in a sample of Italian permanent workers belonging to different occupational sectors, comparing women and men. Gender may be considered a possible moderator of the relationship between one partner’s stressors and the other partner’s outcomes because of differences between men and women in the traditional role requests and expectations and in their way to react to events happening to their family members (Westman, 2001).

## Job Insecurity and Job Satisfaction

The present study considered job insecurity as a subjective perception resulting from an individual’s interpretation of his/her current work condition that reflects the degree to which workers consider their jobs to be vulnerable. In fact, job insecurity is related to concerns about the maintenance of one’s job (Sverke and Hellgren, 2002) that is the employee’s fear to lose his/her occupation and to become unemployed (De Witte, 1999).

Several studies and meta-analyses (Sverke and Hellgren, 2002; Keim et al., 2014; Lee et al., 2018) focused on antecedents of job insecurity, at organizational and individual levels. In particular, microeconomic and social environments, such as labor market characteristics, organizational change and several organizational practices and conditions are potential determinants of job insecurity, in addition to subjective characteristics of the individual.

In literature, job insecurity is recognized as a powerful stressor (Sverke et al., 2002; Cheng and Chan, 2008; De Witte et al., 2016; Lee et al., 2018) that can have detrimental effects on mental and physical health (Hellgren and Sverke, 2003; Silla et al., 2005, 2009; De Witte et al., 2016), psychological well-being or burnout and emotional exhaustion (Mauno et al., 2005; De Cuyper et al., 2012; Kinnunen et al., 2014; Giunchi et al., 2016) as well as job attitudes (Vander Elst et al., 2014) and work performance (Cheng and Chan, 2008; Piccoli et al., 2017).

Studies that considered the negative association of job insecurity with job satisfaction (Hellgren et al., 1999; Urbanaviciute et al., 2015) are well established and provided convergent evidence. Job satisfaction is an emotional state that results from the assessment of an individual’s job experience (Locke, 1976). Research has demonstrated that job insecurity is negatively associated with job satisfaction (Sverke et al., 2002), underlining the critical role of job insecurity in relation to organizational well-being. For example, in the studies of Sverke et al. (2002) and Cheng and Chan (2008), the meta-correlation with job satisfaction is nearly twice the one with mental well-being or physical health: this suggests that job insecurity is particularly related to decreased working well-being (e.g., job satisfaction). Heaney et al. (1994) considered a sample of United States car manufacture workers investigating the consequences of job insecurity and found that protracted job insecurity negatively predicted job satisfaction over time. Similar results were obtained later by Lim (1996), and Preuss and Lautsch (2002). More recently, both Callea et al. (2017) and Chirumbolo et al. (2017) surveyed different samples of Italian workers finding significant negative effects on job satisfaction, also distinguishing between qualitative and quantitative job insecurity as separate, albeit related, predictors. Thus, we propose that:

Hypothesis 1: Focal person’s job insecurity will be negatively associated with his/her job satisfaction.

## Family Life Satisfaction: Spillover and Crossover Effects

Before focusing on available evidence and moving toward our study hypotheses, a theoretical premise is needed, in that we refer to the spillover and crossover hypotheses. Over 40 years ago Kanter (1977), arguing that “occupations contain an emotional climate as well that can be transferred to family life. A person’s work and relative placement in an organization can arouse a set of feelings that are brought home and affect the tenor and dynamics of family life” (p. 47), gave rise to the so-called spillover theory, that is a worker’s experience on the job carry over into his or her non-work experience, and vice versa (Larson et al., 1994). As Frone et al. (1992) pointed out, spillover hypothesis is only one among other hypotheses (e.g., compensation, segmentation, and congruence) trying to explain the intertwinement between work and family domains, although it received stronger scholarly attention and support so far (Edwards and Rothbard, 2000; Heller and Watson, 2005). Still these authors argued that multivariate studies examining the role of third-party variables, namely domain specific stressors, affecting originating domain and receiving domain satisfactions were needed in order to provide stronger empirical support to the spillover hypothesis. Among those variables, job insecurity can play a fundamental role, given its potential detrimental impact (see above) and its perduring preeminence among job stressors due to economic and organizational reasons.

The crossover effect is the process for which psychological well-being (or its absence) is passed from one individual to another (Westman, 2001). Westman (2001, p. 717) defined crossover as “the reaction of individuals to the job stress experienced by those with whom they interact regularly.” The main research area in which the crossover effect is studied is work–family interference in order to understand how experiences lived in work and family “cross over” from one person to his/her partner (Parasuraman and Greenhaus, 2002). Some studies have been conducted also in order to investigate crossover effect in leader–follower relationship (Ten Brummelhuis et al., 2014). Scholars confirmed crossover effects regarding burnout (Bakker and Schaufeli, 2000), marital dissatisfaction (Westman et al., 2004), and work–family conflict (Westman and Etzion, 2005).

In regards to the process that underlines the crossover effect, Bakker and Demerouti (2013) identified two steps: firstly, the individual’s working experiences are transferred to the family field, and secondly, behaviors and emotions in the family field are conveyed to significant others. The transmission may be considered a “contagion process” (Bakker and Schaufeli, 2000) meant as a mutual reaction toward the other partner. Moreover, Westman (2001) specified that the transfer of experiences from one partner to another one could take place through two main mechanisms: an affective and a behavioral crossover. The first mechanism is related to empathy: an individual in an intimate relationship imagines the condition of his/her partner and, doing so, can experience his/her feelings. The second mechanism is related to a series of behaviors (coping, undermining behavior, and social support) that one partner puts in place to deal with the source of stress and that may have a negative impact on the other one.

The crossover model was initially developed by Westman (2001) for strain feelings (e.g., burnout and depression), but other researchers studied also the crossover of positive feelings (e.g., engagement or flow; Bakker and Demerouti, 2007; Bakker et al., 2009). In this study, we took into account the effect of job insecurity on both focal person’s and his/her partner’s family life satisfaction, by the mediation of job satisfaction (of focal person). The negative effect of job insecurity on outcomes pertaining to other life fields (e.g., family life) have been examined to a lesser extent and in an erratic way, providing not definitive evidence.

We anticipated that research examining the impacts of job insecurity on the family domain is far from returning univocal and coherent evidence. Stewart and Barling (1996) found that job insecurity was negatively correlated with job satisfaction; moreover it had no direct effect on parenting style or children’s behavior in Canadian elementary school. A study, which examined also crossover effects (Voydanoff and Donnelly, 1986), showed that job insecurity of fathers was positively linked to the amount of children’s problems as observed by mothers. Job insecurity has been found to be directly related to amplified marital tension (Hughes and Galinsky, 1994) and reduced marital satisfaction of both partners (Larson et al., 1994). Later, Mauno and Kinnunen (1999b), arguing that the processes by which specific job stressors affect marital functioning had not yet been sufficiently specified, found that job insecurity impaired marital satisfaction, although through the intervening role of mediating variables, thus opening the way to the examination of potential mediators. They also examined crossover effects although finding inconsistent results. However, other studies (Riley and Eckenrode, 1986; Belle, 1987) suggested that empathy a wife feels for her partner may suggest she frequently feels his displeasures as if they were her own, resulting in crossover effects of satisfaction and dissatisfaction. Job insecurity experienced by an employee affects the family members by impairing their well-being, satisfaction, or performance due to the fact that an employee transfers his/her job-related worries to his/her spouse/partner or children (Mauno and Kinnunen, 1999b; Westman et al., 2001; Zhao et al., 2012; Kinnunen et al., 2013). Based on such evidence, we propose that:

Hypothesis 2a: Focal person’s job insecurity will be negatively associated with his/her family life satisfaction (spillover effect).
Hypothesis 2b: Focal person’s job insecurity will be negatively associated with family life satisfaction of his/her partner (crossover effect).

Finally, as already anticipated in previously cited studies (e.g., Mauno and Kinnunen, 1999b), it is plausible to expect that the negative relationship of job insecurity with the family domain is mediated by some intervening variables, job satisfaction in our case. It derives that closer attention should be devoted in examining the relationship between job and family satisfaction. Frone et al. (1994) contrasted the spillover hypothesis (i.e., job satisfaction causes family satisfaction) against the congruence hypothesis (i.e., the relationship between job and family satisfaction is characterized best as non-causal), finding support for this second one. Positive and negative long-term spillover effects between marital satisfaction and discord and job satisfaction through a 12-year panel survey have been found (Rogers and May, 2003). Heller and Watson (2005), through a diary study, highlighted a positive relationship between job satisfaction measured in the afternoon and marital satisfaction measured in the evening, which, in turn, predicted job satisfaction the following afternoon. Therefore, we hypothesized that:

Hypothesis 3: The negative relationship between job insecurity and family life satisfaction of both focal person (a) and his/her partner (b) will be mediated by job satisfaction.

## Gender Differences

The study investigated potential gender differences with an exploratory perspective (without formulating specific hypotheses) and tested the three above mentioned study hypotheses separately for women and men. Indeed, previous studies found gender differences in crossover and spillover effects involving job insecurity; nevertheless, results seem not to be fully consistent (Mauno et al., 2017). According to Westman (2001), differences between women and men may be found in several aspects: (1) in the way they react to events happening to the other partner; (2) in the involvement level in family affairs; (3) in demands and expectations traditionally expected. Women are more involved in family activities and concerns and seem to be more vulnerable compared with men to stressors affecting their partners. For these reasons, Westman (2001) considered gender as a possible moderator of the relationship between one person’s stress and his/her partner’s strain.

Moreover, research related to perceptions of job insecurity and gender differences reported ambiguous results. Some studies have not observed gender differences (e.g., Berntson et al., 2010; Giunchi et al., 2016), others have highlighted that women experienced more job insecurity than men (e.g., Mauno and Kinnunen, 2002; Emberland and Rundmo, 2010). The general critical situation in the labor market for women could explain higher level of job insecurity and/or more negative job insecurity related consequences for them compared with men (Keim et al., 2014). On the other side, when studies underlined that men perceive more job insecurity this result could be explained according to the gender role theory, suggesting that family roles are more significant to women’s identity, while work roles are more fundamental to men’s identity (Barnett et al., 1995). Therefore, particularly in Italy, men may be more vulnerable to job insecurity and job loss, since they consider themselves, as the society does, as the “breadwinner” of their own families, while financial matters are considered a secondary responsibility for women (Ghislieri and Colombo, 2014).

In their review, Mauno et al. (2017) reported differences between women and men in the consequences of job insecurity on family-related outcomes. For example, Kinnunen and Mauno (1998) showed that job insecurity decreased work–family conflict only for women. In a longitudinal study a prolonged effect of job insecurity on negative work spillover into parenthood have been found only for women (Mauno and Kinnunen, 1999a). More recently, Richter et al. (2010) showed a mediation of workload in the relationship between job insecurity and work–family conflict only for men. Ford et al. (2007) in their meta-analysis found a moderation of gender in the direct relationship between job stress and family satisfaction, which was stronger for men.

## Materials and Methods

### Ethics Statement

This study used a self-report questionnaire to involve human individuals; the procedure was applied in accordance with the standards of the national law of data treatment followed by the University of Turin and the University of Campania (Italy). The method did not imply medical or other kind of practices that could cause discomfort to involved persons, who were all adult healthy subjects anonymously involved; therefore, further ethical consent was not needed according to the Institutions. Helsinki Declaration (World Medical Association, 2001) and the Italian data protection law (Legislative Decree No. 196/2003) have been respected in the implementation of the study. Participants agreed to be involved voluntarily and without compensation; anonymity was respected in both data collection and analyses. Agreeing to fill in the questionnaire, participants provided their informed consent. Information about study aims, data treatment and anonymity, as well as instructions to complete the questionnaire, were provided in a cover letter.

### Samples and Procedures

Participants were contacted through a convenience sampling procedure; a total sample of 344 Italian heterosexual couples filled out a self-report questionnaire. Questionnaires were distributed within organizations that agreed to participate to the survey by trained researchers, leaving to their employees the decision to participate or not. Employees received a sealable envelope (in order to further protect their privacy) containing a copy of the questionnaire and a letter of research presentation (see above). The questionnaire had two sections: the first one was fill out by participants, who all were permanent workers, and the last one by their partners. Participants and their partners were instructed to complete the questionnaire independently of one another.

Among participants, 183 were female (53.2%) and 161 were male (46.8%). Female participants had at least one child in the 77.6% of the cases; among them 29.5% had one child, 51.8% had two children, 18.7% had three or more children. The level of education was bachelor’s, master’s degrees or higher for the 50.8%, 39.9% had finished high school, the remaining 9.3% had a lower level. The average age was 43.24 years (*SD* = 9.55; min = 26; max = 64). Among women, most of them declared to have a full-time job (71.6%). They were employed in a range of different occupational sectors: 48.1% health, 16.9% private services, 14.2% industry, 9.3% education and research, 4.4% commerce, 7.1% other. Participants worked, on average, 34.74 h per week (*SD* = 9.45; min = 6; max = 60). Mean job tenure was 15.97 years (*SD* = 9.68; min = 1; max = 39). Moreover, organizational seniority was, on average, 13.78 years (*SD* = 9.28; min = 1; max = 37). Among women’s partners, 47.5% had achieved high school, 37.2% a bachelor’s, master’s degrees or higher educational level, 15.3% had a lower level. As for their professional contract, 61.7% had a permanent full-time job, 9.8% had a permanent part-time job, 8.7% had a fixed-term job, 6.6% had other type of job contracts, 13.2% had not specified other situations.

Male participants had at least one child in most of the cases (82.0%); among them 30.8% had one child, 45.9% had two children, 23.3% had three or more children. The level of education was high school for the 51.6%, bachelor’s, master’s degrees or higher for 42.8%, the remaining 5.6% had a lower level. The average age was 44.23 years (*SD* = 9.05; min = 26; max = 67). Among men, most of them declared to have a full-time job (80.7%). They were employed in a range of different occupational sectors: 49.7% health, 32.9% industry, 11.2% private services, 2.5% commerce, 1.9% education and research, 1.8% other. Male participants worked, on average, 37.59 h per week (*SD* = 10.10; min = 6; max = 60). Mean job tenure was 16.36 years (*SD* = 9.80; min = 1; max = 38). Moreover, organizational seniority was, on average, 13.69 years (*SD* = 8.76; min = 1; max = 38). Among men’s partners, 57.8% had achieved high school, 30.4% a bachelor’s, master’s degrees or higher educational level, 11.8% had a lower level. As for their professional contract, 38.5% had a permanent full-time job, 19.3% had a permanent part-time job, 19.3% had a fixed-term job, 8.7% had other type of job contracts, 14.2% had not specified other situations.

### Measures

*Family life satisfaction* of both the respondent and his/her partner was measured by five items (Kobau et al., 2010) with a 7-point Likert scale, from 1 = “Completely disagree” to 7 = “Completely agree” (e.g., “I am satisfied with my family life”). Cronbach’s alpha on the whole sample of this study was 0.94.

*Job satisfaction* was detected by five items of the Copenhagen Psychosocial Questionnaire (Pejtersen et al., 2010) using a 5-point Likert scale, from 1 = “Very unsatisfied” to 5 = “Very satisfied” (e.g., “Indicate your satisfaction about… physical working conditions”). Cronbach’s alpha on the whole sample was 0.90.

*Job insecurity* was measured by four items of the ((s))De Witte (2000) job insecurity scale applying a 5-point Likert scale, from 1 = “Completely disagree” to 5 = “Completely agree” (e.g., “I feel insecure about the future of my job”). Cronbach’s alpha on the whole sample was 0.87.

### Data Analysis

The statistics software IBM SPSS 24 was used to perform descriptive data analyses, Pearson correlations to detect relationships between variables, and Cronbach’s alpha coefficient to verify scales’ reliability in the whole sample and separately in female and male samples. Moreover, analysis of variance through *t*-test for independent samples was calculated in order to investigate potential differences between variables’ means of the female and male samples.

In order to test study hypotheses simultaneously in the two samples, Mplus 7 (Muthén and Muthén, Muthén and Muthén) was applied to test a multi-group full structural equation model (SEM) using Maximum Likelihood (ML) as estimation method. The following goodness-of-fit criteria were considered in order to assess models (Bollen and Long, 1993): the χ^2^ goodness-of-fit statistic; the Comparative Fit Index (CFI); the Tucker Lewis Index (TLI); the Root Mean Square Error of Approximation (RMSEA); and the Standardized Root Mean Square Residual (SRMR). Finally, in order to verify whether mediation effects were significant, we applied bootstrapping procedure (Shrout and Bolger, 2002).

## Results

Correlations between study variables and scales’ internal consistency are shown in **Table 1** for female and male samples. α values ranged between 0.86 and 0.95, respecting the cut-off of 0.70 (Nunnally and Bernstein, 1994). As for correlations, they showed the expected directions in both groups.

**Table 1:** Means, standard deviations, Cronbach’s alpha, and correlations among the study variables for female (*N* = 183) and male (*N* = 161) groups.

	Female sample		Male sample					
	*M*	*SD*	*M*	*SD*	1	2	3	4
1. Family life satisfaction	4.92	1.40	4.93	1.60	*0.93/0.95*	0.74^∗∗^	0.53^∗∗^	-0.22^∗∗^
2. Partner’s family life satisfaction	5.07	1.39	5.12	1.42	0.67^∗∗^	*0.93/0.94*	0.47^∗∗^	-0.17^∗^
3. Job satisfaction	3.43	0.94	3.37	0.99	0.43^∗∗^	0.28^∗∗^	*0.89/0.91*	-0.31^∗∗^
4. Job insecurity	2.24	1.81	2.36	1.13	-0.30^∗∗^	-0.27^∗∗^	-0.47^∗∗^	*0.89/0.86*

*Correlations for the female group below the diagonal; correlations for the male group above the diagonal. Cronbach’s α for female/male samples on the diagonal in italics. ^∗^*p* < 0.05; ^∗∗^*p* < 0.01.*

Job insecurity was negatively correlated in both groups with family life satisfaction (F: *r* = -0.30, *p* < 0.01; M: *r* = -0.22, *p* < 0.01), partner’s family life satisfaction (F: *r* = -0.27, *p* < 0.01; M: *r* = -0.17, *p* < 0.05) and job satisfaction (F: *r* = -0.47, *p* < 0.01; M: *r* = -0.31, *p* < 0.01).

Job satisfaction was positively correlated in both groups with family life satisfaction (F: *r* = 0.43, *p* < 0.01; M: *r* = 0.53, *p* < 0.01) and partner’s family life satisfaction (F: *r* = 0.28, *p* < 0.01; M: *r* = 0.47, *p* < 0.01).

Family life satisfaction and partner’s family life satisfaction are strongly and positively correlated in both groups, as expected (F: *r* = 0.67, *p* < 0.01; M: *r* = 0.74, *p* < 0.01).

Analysis of variance between female and male samples did not show significant differences in the variables’ means.

**FIGURE 1:**
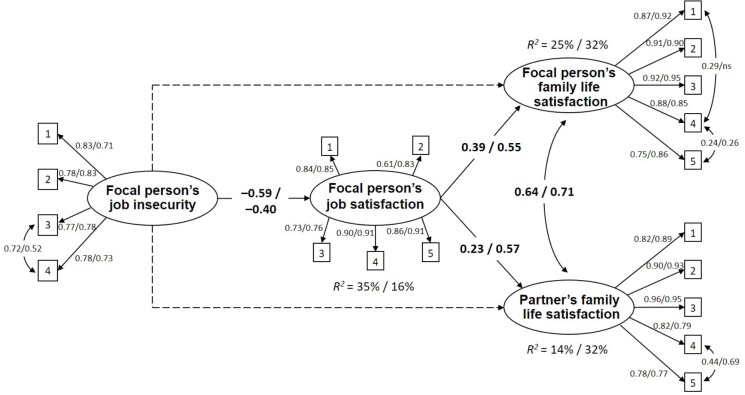
The final model (standardized path coefficients, *p* < 0.05). Results of the multi-group analysis: female/male samples.

The hypothesized multi-group SEM showed a good fit to the data: χ^2^ (312, *N*_Female_ = 183, *N*_Male_ = 161) = 710.03, *p* = 0.00, CFI = 0.94, TLI = 0.93, RMSEA = 0.08 (90% CI [0.07, 0.09]), SRMR = 0.07. The model with standardized parameters for the two samples is depicted in **Figure 1**.

Considering the measurement model, item loadings were acceptable for all variables in the two samples. As for the structural model, job insecurity showed a significant negative association with job satisfaction in both samples (F: β = -0.59, *p* < 0.001; M: β = -0.40, *p* < 0.001). Job insecurity did not present a significant direct relationship with family life satisfaction and partner’s family life satisfaction. In female and male groups job satisfaction had a strong positive relationship with family life satisfaction (F: β = 0.39, *p* < 0.001; M: β = 0.55, *p* < 0.001) and partner’s family life satisfaction (F: β = 0.23, *p* < 0.01; M: β = 0.57, *p* < 0.01). No significant differences between the two samples emerged. The model explained 35% of variance for job satisfaction, 25% for family life satisfaction and 14% for partner’s family life satisfaction in the female sample; and 16% for job satisfaction, 32% for family life satisfaction and 32% for partner’s family life satisfaction in the male sample.

Subsequently, bootstrapping procedure was used to evaluate mediating effects; 2,000 new samples were extracted from the original one and direct and indirect effects were calculated (Preacher and Hayes, 2008). Whether the confidence interval does not include zero it means that the mediation effect is significant. Statistically significant mediated effects are shown in **Table 2**. Particularly, the bootstrapping procedure confirmed that job satisfaction was a full mediator between job insecurity and family life satisfaction in female and male samples, showing indirect effects equal to, respectively, -0.23 and -0.22. Moreover, job satisfaction was a full mediator between job insecurity and partner’s family life satisfaction in female and male samples, showing indirect effects equal to, respectively, -0.13 and -0.23.

**Table 2:** Indirect effects using bootstrapping (2,000 replications).

Indirect effects female sample	Bootstrap			
	Est.	*SE*	*p*	CI 95%
Job Insecurity → Job satisfaction → Family life satisfaction	-0.23	0.06	0.000	(-0.349, -0.107)
Job Insecurity → Job satisfaction → Partner’s family life satisfaction	-0.13	0.07	0.050	(-0.268, -0.002)

**Indirect effects male sample**	**Bootstrap**			
	**Est.**	***SE***	***p***	**CI 95%**

Job Insecurity → Job satisfaction → Family life satisfaction	-0.22	0.07	0.002	(-0.362, -0.080)
Job Insecurity → Job satisfaction → Partner’s family life satisfaction	-0.23	0.07	0.002	(-0.370, -0.086)

*All parameter estimates are presented as standardized coefficients. CI, confidence interval*.

## Discussion

The aim of this study was examining the association between job insecurity and family life satisfaction, with three peculiarities: taking into account the intermediate mediating role of job satisfaction, assessing both partners’ evaluations of family life satisfaction, and differentiating analysis between genders.

Available evidence clearly showed that job insecurity has detrimental effects on well-being and health (De Witte et al., 2016), performance (Cheng and Chan, 2008), and job attitudes (Vander Elst et al., 2014). More in detail, several studies (Sverke et al., 2002; Callea et al., 2017) reported that job insecurity can negatively affect job satisfaction because, through the anticipation of job loss (thus acting as a powerful stressor), it can decrease the pleasant feelings associated with his/her own job. Based on such evidence, we anticipated that a negative association would have existed between job insecurity and job satisfaction. Results from multi-group SEM provided strong support allowing to accept Hypothesis 1. In particular, job insecurity decreased levels of job satisfaction both among male (β = -0.40) and female (β = -0.59) respondents.

Hypothesis 2 postulated that job insecurity would have been negatively associated with family life satisfaction of focal person (a) and his/her partner (b). In doing so, two prominent work–family theories were concerned: the spillover (Kanter, 1977) and the crossover (Westman, 2001) theories. The spillover theory was concerned in regards to Hypothesis 2a, that is job insecurity would have been associated with reduced family life satisfaction, given that negative individuals’ experiences on the job can carry over into his/her non-work experience, and vice versa. Some studies have already provided partial converging evidence (Hughes and Galinsky, 1994; Mauno and Kinnunen, 1999b). The crossover theory was instead concerned to explain the expected negative association between focal person’s job insecurity and his/her partner’s family life satisfaction (Hypothesis 2b), given that individual feelings associated with experiences on one domain can be transmitted to individual’s significant others and affect them. Also in this case, partial (Larson et al., 1994) and contrasting (Mauno and Kinnunen, 1999b) evidence is available. Results from multi-group SEM do allow to partially accept such hypotheses given that no direct associations were present but mediated ones. In fact, deeper examination (see Hypothesis 3) showed that significant indirect effects were present for both male and female respondents (see below for details).

As anticipated by both sparse evidence (Mauno and Kinnunen, 1999b) and theory (Bakker and Demerouti, 2013), it is possible that the spillover and crossover effects of specific job stressors (i.e., job insecurity) may be mediated by intermediate variables. Therefore, Hypothesis 3 stated that the negative effect of family life satisfaction (both focal person’s and his/her partner’s) regressed on job insecurity would have been mediated by job satisfaction. Said differently, fear of losing one’s own job was not expected to impact directly on decreased family life satisfaction, but lowered job satisfaction (as a result of higher job insecurity) could reflect in impaired satisfaction about one’s own and his/her partner’s satisfaction about family life. Results from multi-group SEM provided strong support for Hypothesis 3, which thus can be accepted. In particular, job satisfaction mediated the effect of job insecurity on family life satisfaction (indirect effects: *F* = -0.23, *M* = -0.22) and on partner’s life satisfaction (indirect effects: *F* = -0.13, *M* = -0.23).

As for the exploration of gender role, no differences have been found in this study, which indicated similar patterns for women and men in the spillover and crossover dynamics investigated. Contrary to theories that suggested that women are more likely to experience demands from the family domain spilling over their work role, while for men the opposite situation is more likely (i.e., work domain spilling over the family domain) (Pleck, 1977), our findings contribute to that literature that highlights a greater similarity in the interaction between work and family among genders (Naldini, 2007). Despite some differences still exist, they are decreasing probably because of changes that are involving how men and women perceive their family and work roles, as well as the degree in which these roles are central to their identity. Nowadays, work is considered a central part of their identity also for women, whereas men are more involved in family issues, which are less a women’s prerogative.

### Limitations and Future Studies

A series of limitations need to be acknowledged. First, since a cross-sectional design was used, causality between variables cannot be established (Podsakoff et al., 2012). Moreover, being that only self-report questionnaires were used, common method bias may have partly inflated our results (Conway, 2002). Future studies should recur to longitudinal or diary approaches in order to examine the causal relationship between variables over time, considering also other-reported ratings (such as colleagues or supervisors for the workplace domain).

A second limitation is the use of the convenience sampling procedure that limits results’ generalizability. Despite the sample was a heterogeneous one, the majority of participants had at least one child and a high educational level; this limits the chance to generalize findings to other groups. Moreover, the study involved inter-gender couples; therefore, results are not generalizable to same-gender couples. As regards the procedure, instructions clearly requested each partner to fill out the questionnaire separately; nevertheless, we did not have control whether partners really filled out the questionnaires alone without comparing answers.

Finally, future studies should further examine the dynamics intertwining perceived job insecurity and family life satisfaction of both partners, through the mediation of job satisfaction, considering also buffer effects; particularly, the role of personal resources, such as core self-evaluations, psychological capital, Big Five dimensions, or generic coping strategies, job crafting and employability could be investigated (Silla et al., 2009; Ingusci et al., 2016; Mauno et al., 2017). Moreover, from an opposite point of view, future research should also investigate the crossover of family stressors to the work domain, since family stressors experienced by one individual might be transmitted to the job-related well-being and behavior of his/her partner (Mauno and Kinnunen, 1999b).

## Conclusion and Practical Implications

The present study may contribute to literature mainly for three reasons: (1) it highlighted a spillover effect of job insecurity on the family domain; (2) it found this effect for both the individual and his/her partner confirming a crossover effect; (3) in a working environment where stable jobs are becoming progressively less frequent than temporary ones (Allvin et al., 2013), it confirmed the need to investigate effects of job insecurity also for those workers with permanent contract.

Interventions at the level of Human Resources policies and practices should develop the necessary mechanisms to reduce the perception of job insecurity, considering that helping employees to decrease or tolerate job insecurity may improve their job satisfaction and have certain benefits also for their families. Job insecurity is often increased by the lack of clear information about what workers can expect in their near future (Abildgaard et al., 2017); therefore, organizations may improve their communication system and guarantee clear, objective and timely information, and be available to answer to any requests for clarification about future prospects of both the company and employees (Robinson and Morrison, 2000; Sora and Höge, 2014). Moreover, in case of organizational restructuring or change, which are prevalent today, authors suggested honest, open, and timely communication (Mauno et al., 2017) and a proactive approach with high level of employees’ involvement and participative decision making (Abildgaard et al., 2017) in order to outbalance perception of job insecurity.

Job insecurity issue may be addressed also at the individual level. Particularly, career practitioners may offer counseling and coaching interventions in order to strength workers’ ability to deal with occupational changes, also improving their employability. In addition, life-designing interventions could support individuals in developing adaptive and flexible responses to possible occupational transitions and changing tasks (Savickas, 2012).

Knowledge on crossover between partners may also be useful for both individuals and organizations (Bakker et al., 2005). The former may get a better understanding about their own and their partners’ attitudes and feelings, information that could be used to address potential needs of the couples. The former may use the knowledge on crossover in order to foster working conditions able to enhance job satisfaction, positive experiences at work and, as a consequence, at home. Furthermore, interventions to develop employees’ coping strategies could be also recommendable in order to provide employees with adequate tools for dealing with the job insecurity experience (Sora and Höge, 2014). Particularly, psychological detachment after work, which has been indicated a good recovery strategy for work–family balance (Derks and Bakker, 2014) and well-being (Meijman and Mulder, 1998; Sonnentag and Fritz, 2007), could be effective against job insecurity (Kinnunen et al., 2010) and may be fostered by engaging in positive activities with family during off-job time.

## Author Contributions

FE, MM, ALP, PS, and CG made a contribution to the present study. ALP and CG designed the research and collected the data. FE and MM carried out data analysis and interpretation. FE, MM, ALP, and CG wrote the manuscript receiving substantial input from PS. All authors approved the final version of the manuscript for submission and agreed to be accountable for all aspects of the work.

## Conflict of Interest Statement

The authors declare that the research was conducted in the absence of any commercial or financial relationships that could be construed as a potential conflict of interest.
