# Corrigendum: A Crossover Study From a Gender Perspective: The Relationship Between Job Insecurity, Job Satisfaction, and Partners' Family Life Satisfaction

**DOI:** 10.3389/fpsyg.2018.02055

**Published:** 2018-10-25

**Authors:** Federica Emanuel, Monica Molino, Alessandro Lo Presti, Paola Spagnoli, Chiara Ghislieri

**Affiliations:** ^1^Department of Psychology, University of Turin, Turin, Italy; ^2^Department of Psychology, University of Campania “Luigi Vanvitelli”, Caserta, Italy

**Keywords:** job insecurity, job satisfaction, family life satisfaction, crossover, spillover, permanent workers

In the original article, we neglected to include as a funding statement:

Funding was received from Alessandro Lo Presti's institution, University of Campania “Luigi Vanvitelli”, Programma VALERE.

The authors apologize for this error and state that this does not change the scientific conclusions of the article in any way. The original article has been updated.

## Conflict of interest statement

The authors declare that the research was conducted in the absence of any commercial or financial relationships that could be construed as a potential conflict of interest.

